# A Rare Cause of Thrombotic Thrombocytopenia Purpura- (TTP-) Like Syndrome, Vitamin B12 Deficiency: Interpretation of Significant Pathological Findings

**DOI:** 10.1155/2019/1529306

**Published:** 2019-03-18

**Authors:** Morgan Bailey, Travis Maestas, Robert Betancourt, Dalia Mikhael, Hani M. Babiker

**Affiliations:** ^1^Department of Internal Medicine, University of Arizona, Tucson, AZ, USA; ^2^Department of Internal Medicine, Banner University Medical Center-Tucson, Tucson, AZ, USA; ^3^Department of Hematology-Oncology, University of Arizona Cancer Center, Tucson, AZ, USA

## Abstract

Thrombotic thrombocytopenia purpura (TTP) is a hematological emergency that requires rapid assessment followed by prompt initiation of therapy due to high mortality associated with delayed treatment. TTP has many causes including heritable syndromes, ADAMTS13 deficiency, and drugs-related etiologies. Profound vitamin B12 deficiency can, in rare cases, mimic TTP in presentation, and since plasmapheresis can be of limited benefit, prompt diagnosis is necessary for accurate treatment with B12. Therefore, careful analysis of all clinical signs, symptoms, and labs must be assessed. We report a patient who presented with a diagnosis of TTP, and repeat assessment confirmed a diagnosis of sever vitamin B12 (B12) deficiency with pancytopenia who was appropriately treated with B12.

## 1. Introduction

TTP is characterized by a microangiopathic hemolytic anemia (MAHA), thrombocytopenia, and end-organ dysfunction which requires accurate diagnosis and plasmapheresis [1]. With an incidence of 4 cases in 100000, TTP can have multiple complications including rapid progression to end-organ dysfunction and death [1, 2]. However, it is not uncommon for other conditions, such as vitamin B12 deficiency, to present in a similar fashion, with thrombocytopenia and microangiopathy. Hence, it is important to keep in mind this rare syndrome in addition to other differential diagnoses including disseminated intravascular coagulation (DIC), hemolytic-uremic syndrome (HUS), and autoimmune hemolysis as they can often be mistaken for TTP [2, 3]. Because the results of ADAMTS13 are not readily available at time of presentation, plasmapheresis is often initiated if there is a suspicion for TTP. The case below illustrates such an example in which the patient presented with pancytopenia and microangiopathy concerning for TTP. Emergent plasmapheresis was initiated, and upon further workup, the patient symptoms were attributable to a severe vitamin B12 deficiency.

## 2. Case Presentation

A 70-year-old Caucasian woman with no past medical history presented to her local care facility after sustaining a mechanical fall the day prior to presentation. She endorsed progressive fatigue, weakness, and dyspnea on exertion in addition to symmetric, bilateral lower extremity numbness for two months. Per her husband, she had become progressively more confused over the past week in addition to endorsing new onset jaundice and scleral icterus.

**Figure 1:**
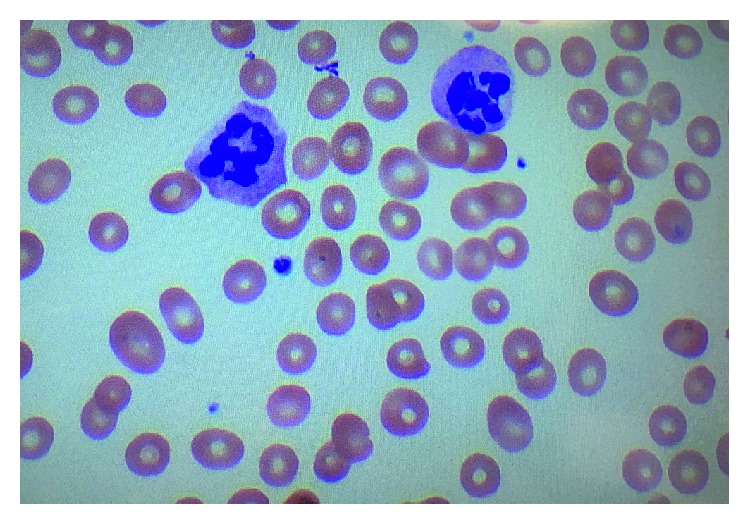
Hypersegmented neutrophils and hypochromic erythrocytes characteristic of a megaloblastic anemia. H&E stain. Magnification: 400x.

**Figure 2:**
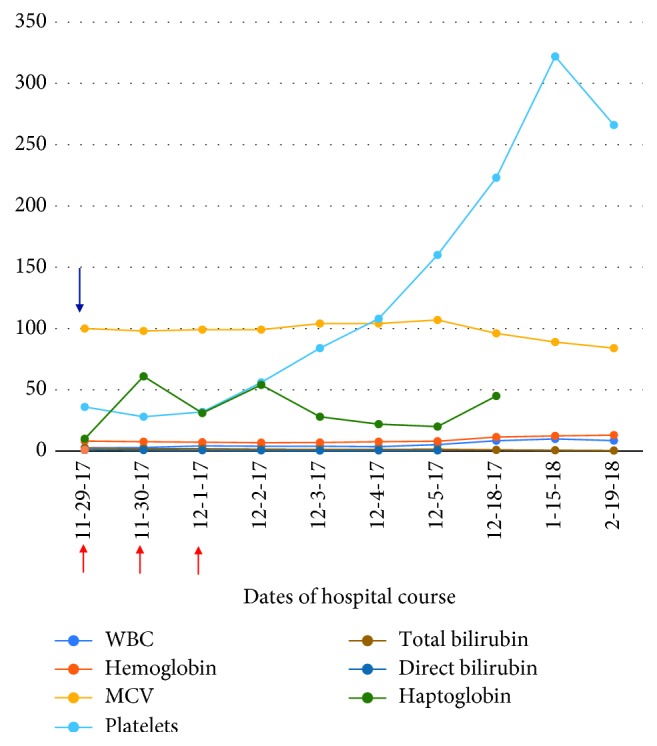
Significant lab values throughout hospital course and three months after initial therapy. Red arrows indicate the dates in which the patient underwent plasmapheresis. The blue arrow indicates the date in which B12 replacement was initiated.

On initial presentation, she was confused and dyspneic. Lab work was significant for a severe anemia with a hemoglobin of 5.7 g/dL (12–16 g/dL). She received four units of pRBCs and was subsequently transferred to our care facility for further evaluation. On presentation, temperature was 36.3°C, pulse 70 bpm, blood pressure 105/57 mmHg, respirations 18, and oxygen saturation of 100% on room air. Physical exam findings were significant for mild scleral icterus and palpable splenomegaly. The patient was initially oriented to person only; however, the remainder of the physical exam, including the neurological exam, was unremarkable without motor or sensory deficits on initial presentation. Initial laboratory studies were significant for pancytopenia with WBC of 1.8 K/mm^3^ (4.0–11.0 K/mm^3^), hemoglobin of 8.3 g/dL (12–16 g/dL), MCV of 103 fL (78–100 fL), RDW 24.7% (11.0–15.0%), haptoglobin of <10 mg/dL (43–212 mg/dL), and platelets of 44 K/mm^3^ (130–450 K/mm^3^) (Table 1). Direct antiglobulin test was negative. Her chemistry profile was significant for a total bilirubin of 3.7 mg/dL (0.2–1.3 mg/dL) with a direct bilirubin of 1.3 mg/dL (0.1–0.5 mg/dL), and a lactate dehydrogenase of 1908 IU/L. Liver chemistries and serum creatinine were normal. Examination of a repeat peripheral blood smear was significant for hypersegmented neutrophils and moderate ovalocytes (Figure 1). In addition, there was concern for schistocytes on the initial smear prompting concern for TTP. At the time of presentation, ADAMTS13 activity was pending. Initially, the patient underwent plasmapheresis starting on admission, for a total of three days in addition to prednisone. Repeat smear was significant for hypersegmented neutrophils and a macrocytic normochromic anemia without evidence of an increase in schistocytes or microspherocytes to suggest a hemolytic process. Serum B12 level drawn on admission was <150 pg/mL (200–650 pg/L). Serum folate level was normal, and serum methylmalonic acid level was ordered to confirm the diagnosis of B12 deficiency and was significantly elevated at 36090 nmol/L (87–318 nmol/L). Anti-intrinsic factor antibodies were positive. In addition, the patient was found to be hypothyroid with TSH of 13.53 mIU/L (0.4–4.0 mIU/L) and free T4 of 0.58 ng/dL and was started on synthroid at 100 *μ*g daily. The ADAMTS13 result eventually returned within normal limits. Therapy with intramuscular B12 was begun with 1000 *μ*g daily, initiated on the same day as plasmapheresis, for one week followed by 1000 *μ*g weekly for four weeks and then monthly thereafter for life. The patient continued to clinically improve with treatment. Repeat vitamin B12 levels one month after therapy were >2000 pg/mL. CBC values also normalized after therapy (Table 2 and Figure 2). Two months after initial therapy, the patient's confusion had resolved; however, her bilateral lower extremity neuropathy continued to persist until the initiation of gabapentin outpatient.

## 3. Discussion

TTP is an emergent diagnosis and necessitates rapid initiation of plasmapheresis if there is suspicion on initial presentation. Due to the high mortality associated with TTP, plasmapheresis is still performed despite ADAMTS13 results not being immediately available. The list of differential diagnoses for hemolytic anemia is broad and can include thrombotic microangiopathies (TMA), autoimmune hemolytic anemia, drug-induced hemolysis (cephalosporins, dapsone, and levodopa), paroxysmal nocturnal hemoglobinuria (PNH), and other hereditary syndromes [13]. Vitamin B12 deficiency is not often associated with hemolytic anemia; however, upon literature review, there have been multiple cases with similar presentations [14] (Table 2). Vitamin B12 deficiency can cause hemolytic anemia most commonly via intramedullary hemolysis of immature reticulocytes; however, thrombotic microangiopathy is rarely seen [15]. Reticulocytopenia in cobalamin deficiency commonly reflects ineffective erythropoiesis in the setting of bone marrow involvement and intramedullary destruction as opposed to peripheral hemolysis as seen with TTP [15, 16]. This mechanism is initiated by premature death via hemolysis of the developing erythrocyte precursors in the bone marrow and is associated with the laboratory findings of hemolysis including elevated LDH, bilirubin, and decreased haptoglobin [16, 17]. However, very few cases of vitamin B12 deficiency have been linked with MAHA, and previous studies indicate that this may arise from acute hyperhomocystinemia which can lead to microangiopathic hemolytic anemia termed pseudothrombotic angiopathy [18]. Therefore, initial laboratory and pathologic findings can often mimic TTP in severe cases of cobalamin deficiency and in rare cases, such as ours, it can present with microangiopathic hemolysis.

## 4. Conclusion

TTP is a life-threatening microangiopathic hemolytic anemia that can lead to death without timely diagnosis and treatment with plasmapheresis. Due to the high mortality associated with TTP, plasmapheresis should be initiated despite pending ADAMTS13 results. As discussed above, TTP can often mimic other common hemolytic anemias; however, there are few cases where severe vitamin B12 deficiency can result in a hemolytic anemia (Table 2). In addition, pathologic findings of severe B12 deficiency can often mimic hemolysis both biochemically and on initial pathology [4, 18, 19]. In the presentation above, the initial working diagnosis was TTP, and thus plasmapheresis was initiated. After further workup and confirmation with normal ADAMTS13 results, the patient was found to be severely deficient in vitamin B12. As discussed, studies have shown that severe vitamin B12 deficiency can present similarly as a hemolytic anemia [1, 4–12] with similar biochemical profiles, thus leading to the initiation of plasmapheresis. This case is important in recognizing this rather unusual presentation of severe vitamin B12 deficiency. Health providers should always act urgently if TTP is suspected, but as this case points out, it is also important to keep other rare causes of hemolytic anemia, such as severe vitamin B12 deficiency.

## Figures and Tables

**Table 1:** Lab values before and after therapy.

Lab values	On presentation	Three weeks after therapy
Hemoglobin	5.7 g/dL	11.5 g/dL
MCV	103 fL	96 fL
Platelet count	44 K/mm^3^	223 K/mm^3^
White blood cell count	1.8 K/mm^3^	8.4 K/mm^3^
Haptoglobin	<10 mg/dL	N/A
Total bilirubin/direct bilirubin	3.7 mg/dL/1.3 mg/dL	1.0 mg/dL/NA
LDH	1908 IU/L	279 IU/L H
Serum B12 level	<150 pg/mL	>2000 pg/mL

**Table 2:** Case reports of severe vitamin B12 presenting with findings similar to TTP. Cases are organized by age of patient, clinical symptoms at time of presentation, treatment, and outcomes.

Manuscript	Age	Presentation	Treatment	Outcome
Rodrigues et al. [4]	10-month-old F	Two weeks of intermittent vomiting, poor feeding, and decrease in weight percentile from 50% to 5%. Marked pallor, slightly jaundiced appearance, and mild dehydration	Red blood cells transfusion, cyanocobalamin (50 *μ*g subcutaneously, five doses; 100 *μ*g, oral, per day for 1 month), folic acid (5 mg/day), and food diversification	Biochemical and hematologic remission after 1 week and 1 month, respectively. Infant development normalized at 24 months
Tadakamalla et al. [5]	31 yo F	One month of generalized fatigue and bilateral paresthesias of the feet for one week	Plasmapheresis for 4 days followed by IM vitamin B12	B12 def 2/2 pernicious anemia. Complete biochemical response and resolution of symptoms at 4 months
Routh and Koenig [6]	43 yo M	Two-week history of confusion, fever, dyspnea, dizziness, and fatigue associated with diarrhea and hematochezia	3 units FFP, plasmapheresis IM B12	Not reported
Podder et al. [7]	46 yo M	One day history of hematuria and hemoptysis	Plasmapheresis 24 units FFP	Resolution of biochemical parameters. Acquired TTP in the setting of pernicious anemia
Chhabra et al. [8]	52 yo M	Fatigue, loose stools, and weight loss for six months	Intermuscular vitamin B12	Hematological resolution of anemia
Walter et at. [9]	77 yo F	Altered mental status, renal insufficiency, and thrombocytopenia	Plasmapheresis IM vitamin B 12 for life	Resolution of clinical symptoms and anemia 3 weeks after discharge
Merino and Cid [10]	36 yo M	3-4 weeks of asthenia in addition to fatigue and conjunctival pallor	IM vitamin B12 for life	Resolution of clinical and biochemical parameters
Trubin et al. [11]	41 yo F	Two months of fatigue	IM vitamin B12 for 10 days	Resolution of symptoms and correction of anemia
Chapuis et al. [12]	52 yo M	14 days of shortness of breath, general weakness, weight loss, and a sore tongue	2 units pRBCs IM vitamin B12 for life	Resolution of biochemical parameters 9 days after therapy. Resolution of symptoms 6 months after therapy
